# Multi-Response Optimization of Al_2_O_3_ Nanopowder-Mixed Wire Electrical Discharge Machining Process Parameters of Nitinol Shape Memory Alloy

**DOI:** 10.3390/ma15062018

**Published:** 2022-03-09

**Authors:** Rakesh Chaudhari, Parth Prajapati, Sakshum Khanna, Jay Vora, Vivek K. Patel, Danil Yurievich Pimenov, Khaled Giasin

**Affiliations:** 1 Department of Mechanical Engineering, School of Technology, Pandit Deendayal Energy University, Raisan, Gandhinagar 382007, India; rakesh.chaudhari@sot.pdpu.ac.in (R.C.); parth.prajapati@sot.pdpu.ac.in (P.P.); vivekp@sot.pdpu.ac.in (V.K.P.); 2 Department of Solar Engineering, School of Technology, Pandit Deendayal Energy University, Raisan, Gandhinagar 382007, India; sakshum.kphd16@sot.pdpu.ac.in; 3 Department of Automated Mechanical Engineering, South Ural State University, Lenin Prosp. 76, 454080 Chelyabinsk, Russia; danil_u@rambler.ru; 4 School of Mechanical and Design Engineering, University of Portsmouth, Portsmouth PO1 3DJ, UK

**Keywords:** shape memory alloy, Al_2_O_3_ nanopowder, Nitinol, surface morphology, wire electrical discharge machining, Teaching–Learning-Based Optimization algorithm

## Abstract

Shape memory alloy (SMA), particularly those having a nickel–titanium combination, can memorize and regain original shape after heating. The superior properties of these alloys, such as better corrosion resistance, inherent shape memory effect, better wear resistance, and adequate superelasticity, as well as biocompatibility, make them a preferable alloy to be used in automotive, aerospace, actuators, robotics, medical, and many other engineering fields. Precise machining of such materials requires inputs of intellectual machining approaches, such as wire electrical discharge machining (WEDM). Machining capabilities of the process can further be enhanced by the addition of Al_2_O_3_ nanopowder in the dielectric fluid. Selected input machining process parameters include the following: pulse-on time (T_on_), pulse-off time (T_off_), and Al_2_O_3_ nanopowder concentration. Surface roughness (SR), material removal rate (MRR), and recast layer thickness (RLT) were identified as the response variables. In this study, Taguchi’s three levels L_9_ approach was used to conduct experimental trials. The analysis of variance (ANOVA) technique was implemented to reaffirm the significance and adequacy of the regression model. Al_2_O_3_ nanopowder was found to have the highest contributing effect of 76.13% contribution, T_on_ was found to be the highest contributing factor for SR and RLT having 91.88% and 88.3% contribution, respectively. Single-objective optimization analysis generated the lowest MRR value of 0.3228 g/min (at T_on_ of 90 µs, T_off_ of 5 µs, and powder concentration of 2 g/L), the lowest SR value of 3.13 µm, and the lowest RLT value of 10.24 (both responses at T_on_ of 30 µs, T_off_ of 25 µs, and powder concentration of 2 g/L). A specific multi-objective Teaching–Learning-Based Optimization (TLBO) algorithm was implemented to generate optimal points which highlight the non-dominant feasible solutions. The least error between predicted and actual values suggests the effectiveness of both the regression model and the TLBO algorithms. Confirmatory trials have shown an extremely close relation which shows the suitability of both the regression model and the TLBO algorithm for the machining of the nanopowder-mixed WEDM process for Nitinol SMA. A considerable reduction in surface defects owing to the addition of Al_2_O_3_ powder was observed in surface morphology analysis.

## 1. Introduction

A new group of alloys, also known as smart materials, are gaining popularity due to their unique feature of remembering their shape throughout their lifecycle. These are also termed shape memory alloys (SMAs) which mutate back to their original shape on heating after deformation under load [1,2]. In addition, SMAs also possess generic properties such as superelasticity, favorable microstructure, and pseudoelasticity [3,4]. The reversible martensitic phase transformation enables SMAs to generate higher work output and higher stress and strain actuation [5]. Several alloys and their combinations, such as Nickel–Titanium (NiTi), CuAlNi, Au–Cd, etc., have been developed showing shape memory effects [6,7]. Among various applications of SMAs, the most popular uses include the following: biomedical, aerospace, automotive, robotics, etc., in addition to the exploration of newer engineering fields [8,9]. The release of Ni ions in biofluid is prevented by the formation of a protective TiO_2_ layer which is formed from titanium material present in Nitinol [10,11]. This leads to Nitinol`s suitability for biomedical applications. The hardening of Nickel-based alloys is comparatively faster owing to the presence of an austenitic matrix which is the biggest hindrance to the machining ease of alloys [12,13]. Additionally, the pseudoelasticity and higher ductility properties of Nitinol pose a challenge for the machining of Nitinol. Several causes that obstruct the machining of SMAs via conventional methods include excessive time for machining, formation of burrs, excessive tool wear, inferior cutting efficiency, and unacceptable surface quality [14,15]. This makes non-conventional machining techniques fairly suitable for SMAs, especially for Nitinol. To achieve dimensional precision and reasonable surface integrity, surface roughness (SR), high production rate, and thin recast layer thickness (RLT) become mandatory to achieve during the manufacturing of instruments for biomedical applications given the nature of intricacy. Researchers are in pursuit of the same.

Non-contact operation of the wire electrical discharge machining (WEDM) technique, among work material and machining tool (wire), extensively reduces the difficulties of conventional machining methods [16,17]. Complex shape implants and structures are one of the key requirements for biomedical applications [18]. WEDM is an advanced machining process capable of producing complex shape geometries [19]. During the process, serial sparks are formed between tool and component which erodes the little amount of material through melting and vaporization [20,21]. Dielectric fluid is used in the machining zone which helps to remove the eroded material particles [22]. This phenomenon of dielectric flushing and sparking forms a hard and uneven machined surface [23]. Thus, SR becomes a crucial response variable during the WEDM process. The WEDM method involves a multiple and complex control of parameters for obtaining a better surface [24]. For the industry, higher productivity is also of prime importance, in addition to the excellent machined surface which can be achieved by enhancing the material removal rate (MRR) and concurrently decreasing SR and RLT. One of the ways to achieve this is to mix nanopowder in dielectric fluid during the WEDM process.

The current trend suggests that powder-mixed dielectric fluid is most popular among researchers for obtaining optimum parametric settings to achieve multiple objectives [25,26]. A series of research studies using Al, CNT, Cr, etc., as additives have been experimented by researchers with dielectric fluid for the EDM process [27,28]. For this process, different parameters for powder characteristics, including powder concentration, powder size, thermal and electrical conductivity, and powder density, have a significant effect on the process [28]. Anil Kumar et al. [29] implemented Grey–Taguchi’s combined approach for the powder-mixed EDM (PMEDM) process. Improvement in all responses has been found after the addition of powder with dielectric fluid. Prakash et al. [30] studied the effect of Si powder on the EDM process of titanium alloys. Their results depicted the improvement in MRR along with the reduction in tool wear rate (TWR). They also observed reduction in the recast layer thickness (RLT) and reduction in surface defects at Si powder concentration of 4 g/L. Amit et al. [31] analyzed the effect of the PMEDM process by adding Al_2_O_3_ nanopowder along with dielectric fluid for obtaining a better machining output. Both the response variables (MRR and SR) were improved to a large extent with the modified dielectric fluid along with better sparking stability of the nanopowder-mixed EDM (NPMEDM) process. Sahu and Mandal [32] analyzed the influence of Al_2_O_3_ PMEDM for the EDM process of Nimonic-263. They observed improvement in the surface morphology of machined surfaces with the use of Al_2_O_3_ PMEDM. Kumar et al. [33] used Al_2_O_3_ nanopowder to study the performance of the EDM process on MRR and SR. They compared the performance of the Al_2_O_3_ nanopowder with the conventional EDM process and observed an improvement of 44% and 51% in MRR and SR, respectively, with the use of nanopowder. Tan and Yeo [34] observed a substantial reduction in RCL with the use of nanopowder concentration in dielectric fluid. Aiyeshah et al. [35] obtained a minimum SR of 3.107 µm and RCL of 14.92 µm with the use of Si powder for Nimonic-90 superalloy. Pulse-on time (T_on_), pulse-on time (T_off_), current, and amount of Si powder were found to be the most contributing factors for obtaining the desired levels of SR and RLT. Vinay et al. [36] used aluminum oxide powder in EDM machining of Inconel 825. SR of the machined samples improved with the addition of powder concentration. Another study, conducted by Sagar et al. [37], used Al_2_O_3_ powder in EDM for Inconel 718 and reported that the response variables depend on the EDM machining variables. Little effect of Al_2_O_3_ powder was seen on the response variables. It becomes essential to control all the machining variables simultaneously to achieve optimal levels of multiple response variables.

On detailed examination of relevant literature, researchers used different powders for the machining of various alloys. Researchers have given more attention to the improvement of responses such as MRR, SR, and TWR. However, very limited work was carried out on other useful output parameters such as RLT and surface morphology of the machined surface. To the best of our knowledge, the effect of Al_2_O_3_ nanopowder and simultaneous optimization of WEDM parameters using the Teaching–Learning-Based Optimization (TLBO) algorithm for Nitinol SMA has not yet been reported. In the current study, a handful of work considering T_on_, T_off_, and amount of Al_2_O_3_ nanopowder as the input variables along with MRR, SR, and RLT as the response variables of Ni_55.8_Ti SMA has been reported. Experiments were conducted using Taguchi’s L_9_ orthogonal arrays. Analysis of variance (ANOVA) was used to check the adequacy and significance of the variables. Taguchi’s approach has a limitation of attaining only one response variable at a time. In addition, the TLBO algorithm was implemented for simultaneous optimization of output responses of MRR, SR, and RLT. Lastly, the surface morphology of the machined surface was reported by SEM analysis. The authors strongly consider this study to be very useful for industrial applications.

## 2. Preparation of Al_2_O_3_ Nanopowder

The chemical reagents aluminum nitrate nanohydrate (Al(NO_3_)_3_·9H_2_O), citric acid (C_6_H_8_O_7_), triethanolamine (N(CH_2_CH_2_OH)), and ethylene glycol (EG) were purchased from Sigma Aldrich Inc. and were used without further purification. Ultrapure water with resistivity 18.2 MΩ-cm was used throughout the experiments. In a typical process, aluminum nitrate nanohydrate was dissolved in deionized water and stirred at medium speed to obtain a uniform mixture. Subsequently, triethanolamine was added dropwise in the solution. Later, citric acid was slowly added to this solution and stirred at 75 °C for 45 min; the color of the sols changed and the obtained sols were then heated up to 150 °C for 90 min, resulting in the viscous gels. For complete drying, the sol was thermally heated in an inert atmosphere at 600 °C for 3 h to produce Al_2_O_3_ nanopowders. The synthesized Al_2_O_3_ nanopowders were characterized with scanning electron microscopy (Zeiss Ultra 55 at 5 kV), X-ray diffraction (XRD) (Panalytical X’pert Pro with the source of Cu-Kα radiation of 0.154 nm, λ = 1.54 Å and acceleration voltage of 45 kV and 40 mA) in a range from 10 to 90°, and Raman Spectroscopy (using a laser of wavelength 532 nm).

## 3. Experimental Plan and Methods

### 3.1. Experimental Details

In the present study, experimentation was accomplished with Ni_55.8_Ti SMA, 6 mm in diameter. Molybdenum wire was used as an electrode. The chemical composition contains the major elements Ni (55.78%) and Ti as a remainder. Al_2_O_3_ nanopowder was mixed along with EDM oil in a tank with various amounts, as per the experimental plan. The stirrer was used for uniform mixing of Al_2_O_3_ nanopowder in dielectric fluid. This will not allow nanopowder to settle at bottom of the tank. The Al_2_O_3_-mixed dielectric fluid was sprayed through nozzles in the machining zone. Three useful input parameters, namely T_on_, T_off,_ and amount of Al_2_O_3_ nanopowder, were varied during the experimental trials. Experiments were performed by using Taguchi’s L_9_ orthogonal arrays. Slices of 1.5 mm in length were cut from the rod following the experimental matrix generated using Taguchi’s design. Table 1 shows the experimental conditions of the PMWEDM operation. Process parameters and their levels were selected, as per the literature studies, pilot tests, and machining limits. In the current study, MRR, SR, and RLT are considered response variables. Two or more repetitions are mandatory for each trial to assess the experimental error and provide the conditions required to test the hypothesis [38,39]. All the experiments were repeated three times in the current study and the average value of the obtained results was considered for analysis. Minitab v17 was employed to analyze the experimental results.

The MRR was measured (gram/minute) by determining the weight of the machined components, as per Equation (1).

(1) $$MRR=\frac{\left(W_{bm}-W_{am}\right)\times 60\;}{t}$$

where $W_{bm}$ and $W_{am}$ are the weight of the components in grams, and t represents the time in seconds.

The Mitutoyo Surftest SJ-410 model was used to measure the SR by selecting the cut-off length of 0.8 mm. SR values were measured at three locations and the average value was taken for analysis.

RLT and surface morphology of the machined surface were measured by using a FE-SEM. Initially, the machined samples were mechanically polished using different grades of emery paper, and then chemically etched (14 mL HNO_3_, 4 mL HF, 82 mL H_2_O) to avoid burrs. Specimens were then explored for the measurement of RLT through the SEM technique.

### 3.2. Optimization Using TLBO Algorithm

**Figure 1:**
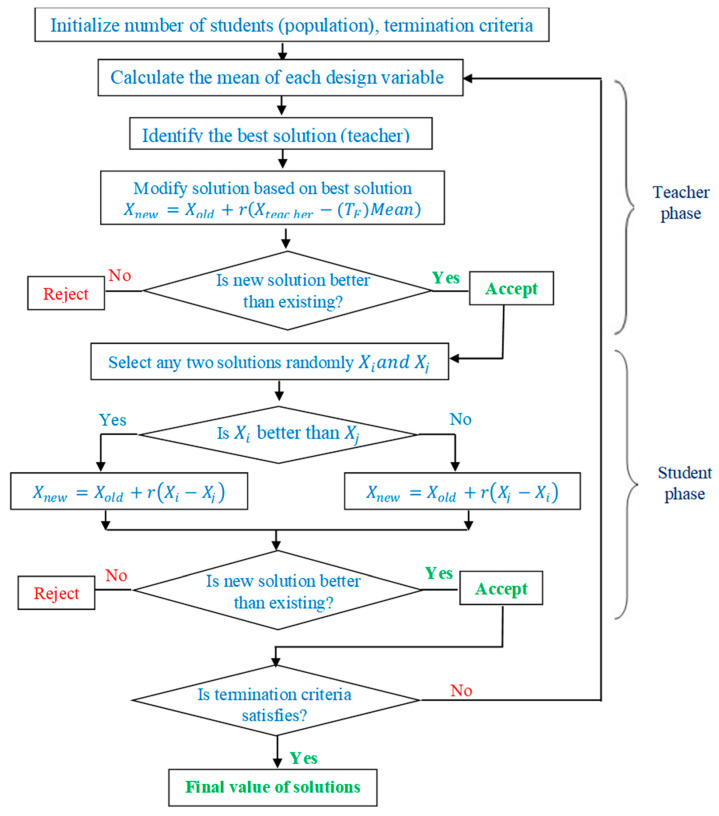
Flowchart of the TLBO algorithm.

For the current study, Teaching–Learning-Based Optimization (TLBO) algorithm is used to obtain the optimum values of the machining parameters. Multi-objective optimization is applied to such problems when there is a trade-off between conflicting objectives. TLBO overcomes the complexity of tuning control parameters and offers ease in the computational time compared to other multi-objective optimization algorithms [40]. It is an easy and efficient algorithm wherein the significance of a teacher on the outcome of the learner is taken into consideration. It adapts the teaching–learning process in a traditional classroom where students learn from teachers to improve their knowledge. Furtherly, students can also interact among themselves to share their knowledge, hence, TLBO involves the following two-phase learning: (a) teacher phase—wherein students learn from teacher; (b) learner phase—wherein students interact among learners [41]. Figure 1 represents the flowchart of the processes involved in the TLBO algorithm.

With this analogy, the TLBO algorithm is developed to obtain good results (objective function) for the given class of students (population) by teaching different subjects (design variables). The result of the student after undergoing the teaching–learning process is as good as the fitness value of an objective function. The best result (solution) from the class is considered as a teacher. A good teacher strives to bring the level of learners to his or her knowledge and eventually increases the mean of the results. However, in practice, not each learner can reach up to a level of the teacher, as it depends on the individual’s capability [42]. If *M_i_* is the mean of the class, then for each *i*th iteration, the solution in the teacher phase will be updated for the existing and new mean which is given as follows:

*Difference_Mean_i_* = *r_i_* (*M_new_* − *T_F_ M_i_*) (2)

where *r_i_* represents a random number generated between 0 and 1, *M_new_* represents the values of design variables corresponding to the teacher, and *M_i_* represents the mean value of design variables considering all students.

*T_F_* = *round* (1) + *rand* (0, 1) (3)

*X_new,i_* = *X_old,i_* + *Difference_Mean_i_* (4)

where *T_F_* is the teaching factor which decides the mean value, either 1 or 2.

In the learner’s phase, learners improve their knowledge through interaction between themselves. If a learner interacts through group discussion and presentations, with the learner having more knowledge, the former ones learn something new. Upon selecting two learners randomly, modification is presented, as per Equations (5) and (6):

*If f*(*X_j_*) < *f*(*X_k_*),
*X_new,j_* = *X_old,j_* + *r_j_* (*X_j_* − *X_k_*) (5)

*Else* *X_new,j_* = *X_old,j_* + *r_j_* (*X_k_* − *X_j_*) (6)

where, *r_j_* represents a random number generated between 0 and 1, and the subscript *i* represents iterations, while *j* and *k* represent the population, where *j* ≠ *k*.

## 4. Results and Discussions

### 4.1. Morphological and Structural Analysis

**Figure 2:**
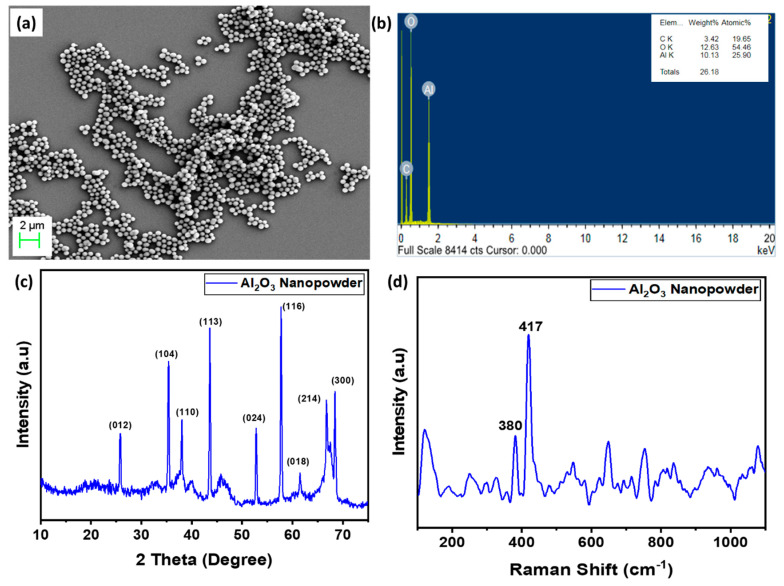
Morphological and structural analysis of as prepared α-phase Al_2_O_3:_ (**a**) FE-SEM, (**b**) EDX, (**c**) X-ray diffraction spectra, and (**d**) Raman Profile.

The morphology and structure of as-synthesized Al_2_O_3_ nanopowder were examined under a field emission scanning electron microscope (FE-SEM), as shown in Figure 2a. It was observed that the nanopowder was in the range of about 100 nm. Further, energy-dispersive X-ray spectroscopy was carried out to determine the elements present in the as-synthesized Al_2_O_3_ nanopowder, shown in Figure 2b. The results confirmed the presence of aluminum and oxygen in the synthesized material and no extra elements were observed other than carbon, which can be attributed to the presence of carbon in carbon tape. In addition, the structural properties of the prepared Al_2_O_3_ were analyzed under X-ray diffraction and Raman Spectroscopy (Renishaw in via Raman Microscope, Pune, India), as shown in Figure 2c,d. The diffraction profile shows peaks at 2θ~68.2°, 66.3°, 57.6°, 52.4°, 43.7°, 37.8°, 35.2°, and 25.3° can be attributed to 300, 214, 116, 024, 113, 110, 104, and 012 planes (JCPDS No 46-1212), confirming the formation of α-Al_2_O_3_ with hexagonal structure [43]. The average crystallite size obtained from peaks was 27 nm using the Debye–Scherrer formula, as shown in Equation (7):

(7) $$D=\frac{0.9\lambda }{\beta Cos\theta }$$

where ‘*β*’ represents full width at half maximum (FWHM), ‘*D*’ represents the crystalline size, ’λ’ represents the wavelength of CuKα radiation, and ‘θ’ represents the angle of diffraction. Further, the Raman profile (Figure 2d) showed characteristic peaks at 378 cm^−1^ and 416 cm^−1^, which were found in agreement with the reported work of Cava et al. [44]. The sharp peaks of the α-phase indicate the well-defined long-range order in corundum and large grain sizes. The powders do not present any impurities, as no extra peaks were observed. The Raman spectra also confirm the formation of α-phase Al_2_O_3_ and were found in agreement with XRD results.

### 4.2. Experimental Results as per Taguchi’s L_9_ OA

The Al_2_O_3_ nanopowder-mixed WEDM process variables, as per the L_9_ Taguchi OA, are shown in Table 2. Experimentally obtained results of the process variables (MRR, SR, and RLT) are also presented in Table 2.

### 4.3. Parametric Effect on MRR

ANOVA can be effectively used to check the relative significance of input parameters on output variables. A 95% confidence level was selected to determine the significance of WEDM parameters, such as T_on_, T_off,_ and Al_2_O_3_ nanopowder, on MRR. Lower P-/higher F-value indicates the larger influence of the machining variable on the selected response [45]. To have a significance of an input variable on the output variable, it is desired to have a *p*-value of less than 0.05 [46,47]. Table 3 illustrates the ANOVA for MRR. All the input variables, such as T_on_, T_off_, and Al_2_O_3_ nanopowder, were having a significant effect on the MRR, as their *p*-value was less than 0.05. The Al_2_O_3_ nanopowder has the highest contribution of 76.13%, followed by T_off_ (17.47%) and T_on_ (7.14%). A small deviation among the R-sq. and Adj. R-sq. signifies the adequacy and fitness of the model [48]. A negligible difference in R-Sq. values of the existing model suggests that it is adequate and fit for estimation of future outcomes of MRR. The regression model for the prediction of MRR is shown in Equation (8), as follows:

(8) $$\mathrm{MRR}\;=-0.0730+0.0471\cdot \mathrm{Current}+0.0111\cdot T_{\mathrm{on}}-0.0143\cdot T_{\mathrm{off}\;}+0.1299\cdot \mathrm{Powder}\;\mathrm{Conc}.$$

**Figure 3:**
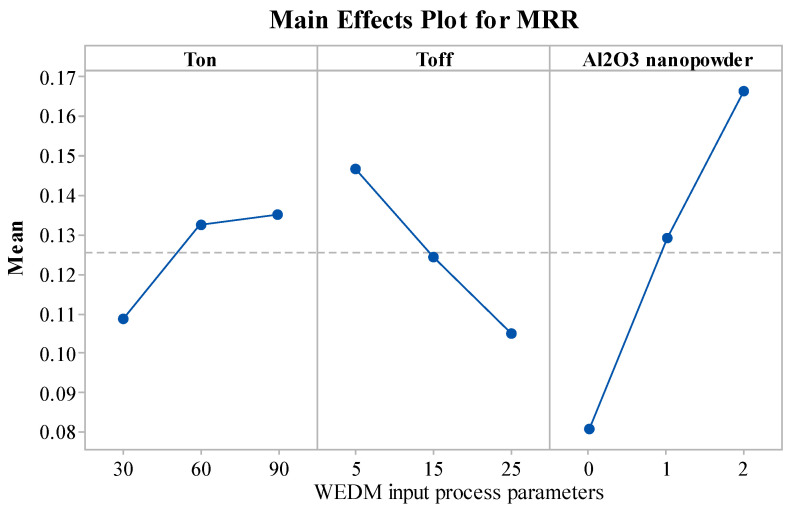
Influence of WEDM variables on MRR.

The significance of the WEDM variables on MRR is illustrated in Figure 3. The MRR value was enhanced with an increase in the concentration of Al_2_O_3_ nanopowder. The MRR has improved substantially with the increase in Al_2_O_3_ nanopowder, as the sparking frequency and thermal conductivity of the dielectric fluid increases [27]. This further increases the rate of erosion of the work material and gives the increase in MRR. From Figure 3, upon increasing T_on_, the value of MRR was improved. As T_on_ increases, discharge energy and spark intensity also increase [49]. Discharge energy gets converted into thermal energy. Thermal energy melts and vaporizes the material from the work surface [50]. Thus, higher thermal energy erodes more material from work, and thereby, increases the MRR. T_off_ was found to have a reverse on MRR, in comparison to T_on_. An increase in T_off_ reduces the intensity of the spark [51,52]. This further reduces the discharge energy, and subsequently, the MRR value also decreases with an increase in T_off_.

### 4.4. Analysis of SR

ANOVA was employed to evaluate the regression coefficients of the model. Table 4 illustrates the ANOVA of SR. A 95% confidence level was selected to determine the significant impact of machining parameters such as T_on_, T_off,_ and Al_2_O_3_ nanopowder on SR. T_on_, T_off_, and Al_2_O_3_ nanopowder were observed as significant contributing factors for SR response as *p*-value of these input factors was found to be less than 0.05. T_on_ was found to be the highest contributing factor (91.88%), trailed by T_off_ (6.36%) and Al_2_O_3_ nanopowder (1.17%). A negligible error of 0.58% was found for SR. A small deviation among the R-sq. and Adj. R-sq. signifies the adequacy and fitness of the model [53,54]. A negligible difference in R-Sq. values of the existing model suggest that it is adequate and fit for estimation of future outcomes of SR. The regression model for the prediction of SR is shown in Equation (9), as follows:

(9) $$\mathrm{SR}\;=3.195+0.2522\;\cdot \mathrm{Current}+0.0565\cdot T_{\mathrm{on}}-0.0588\cdot T_{\mathrm{off}}-0.3550\cdot \mathrm{Powder}\;\mathrm{Conc}$$

**Figure 4:**
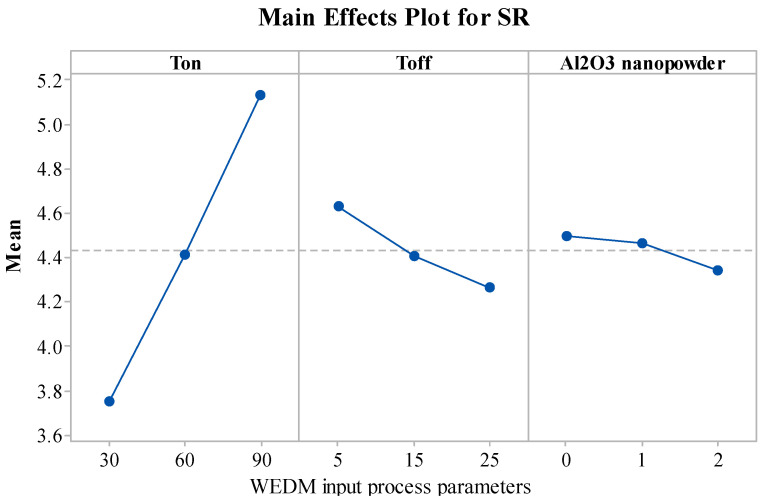
Influence of WEDM variables on SR.

Figure 4 describes the significance of the WEDM variables on the SR response. SR of the machined components was found to follow an increasing trend with a rise in the value of T_on_. The rise in the T_on_ value increases the discharge energy, which in turn increases the thermal energy [55,56]. Due to higher discharge energy and thermal energy, plasma channel pressure increases, impulsive forces are created, and this produces rough and irregular surfaces [56]. Thus, due to the higher thermal energy, SR value increases with an increase in T_on_ value. The declined trend was noticed in SR with an increase in T_off_. An increase in T_off_ decreases the discharge energy and small craters are created [57,58]. This increases the quality of the surface by decreasing the SR value. Another reason for the decreasing SR is that with an increase in the T_off_ value, the flushing of debris gains more time and it removes the unwanted eroded particles from the machined zone [59,60]. From Figure 4, by increasing the Al_2_O_3_ amount, a decrease in the SR was identified. The addition of nanopowder concentration expands the interelectrode gap and also increases the heat dissipation in the dielectric fluid by forming small craters and reducing the plasma heat flux [31,61]. The addition of Al_2_O_3_ nanopowder enhances the flushing of debris from the machining zone. Improved flushing of debris forms small ridges resulting in improved surface quality [27].

### 4.5. Analysis of RLT

Investigation on the effect of the input process parameters of T_on_, T_off,_ and Al_2_O_3_ nanopowder on RLT was carried out by using ANOVA. Specimens were explored for the measurement of RLT through the SEM technique at 1000× magnification. Table 5 illustrates the ANOVA for RLT. All the input variables, such as T_on_, T_off_, and Al_2_O_3_ nanopowder, were having a significant effect on the RLT as their P-value was observed to be less than 0.05. T_on_ was found to be the highest contributing factor (88.3%), trailed by T_off_ (7.85%) and Al_2_O_3_ nanopowder (3.38%). A negligible error contribution of 0.47% was observed for SR. A small deviation among the R-sq. and Adj. R-sq. signifies the adequacy and fitness of the model. A negligible difference in R-Sq. values of the existing model suggest that it is adequate and fit for the estimation of future outcomes of RLT. The regression model for the prediction of RLT is shown in Equation (10), as follows:

(10) $$\mathrm{RLT}\;=3.195+0.2522\;\cdot \mathrm{Current}+0.0565\cdot T_{\mathrm{on}}-0.0588\cdot T_{\mathrm{off}}-0.3550\cdot \mathrm{Powder}\;\mathrm{Conc}$$

**Figure 5:**
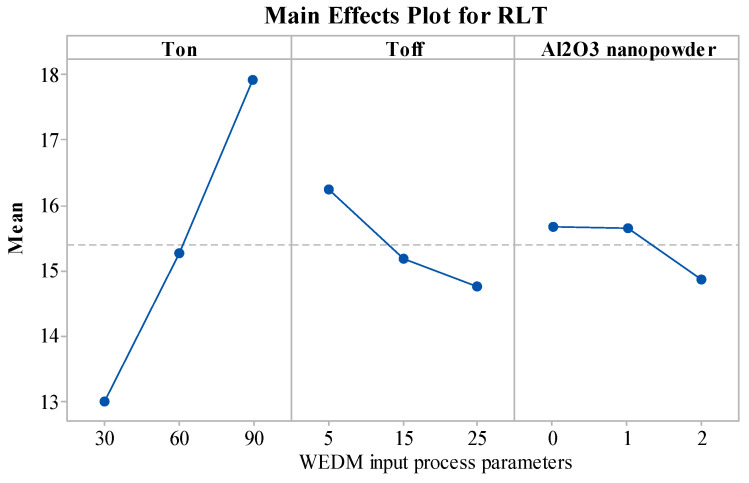
Effect of input process parameters on RLT.

Figure 5 describes the influence of the WEDM process parameters on RLT. The RLT of the machined components was found to follow the increasing trend with a rise in the value of T_on_. An increase in the T_on_ value creates larger dispersive energy, which in turn melts more work material. With the increased value of T_on_, the dielectric is unable to flush the molten metal, due to which it gets deposited on the machined surface [62]. This unflushed molten metal then quenches and re-solidifies by forming a thick RLT [63]. However, with an increase in the value of T_off_, RLT decreases as the melting of the material reduces with the increased value of T_off_. Additionally, debris of work material gain enough time to flush away from the machining zone [64]. This reduces the RLT of the machined zone. From Figure 5, by increasing the Al_2_O_3_ amount, a decrease in RLT was noticed. The addition of nanopowder increases the interelectrode gap and reduces the insulation strength of the dielectric by reducing the energy density [27,64]. This in turn produces a thin RLT, as it largely eliminates the redeposition of debris in the machined zone. Additionally, with the addition of Al_2_O_3_ nanopowder, the spark gap increases. This reduces the discharge energy in the machined zone, and Al_2_O_3_ nanopowder removes the dissipated heat from the zone. It creates shallow craters and forms a thin RLT [34,65].

### 4.6. Optimization Using TLBO Algorithm

TLBO algorithm was executed by considering all responses as positive integers. Following machining, limits were used for the WEDM process parameters during the execution of the algorithms.

(T_on_): 30 µs ≤ T_on_ ≥ 90 µs

(A_off_): 5 µs ≤ T_off_ ≥ 25 µs

(Al_2_O_3_ Conc.): 0 g/L ≤ Al_2_O_3_ Conc. ≥ 2 g/L

Single-objective optimization was carried out for the response parameters to maximize MRR and minimize SR and RLT using the TLBO algorithm. The effect of machining variables T_on_, T_off,_ and Al_2_O_3_ nanopowder was studied on the aforementioned response parameters. Results of the optimization are presented in Table 6. Within the selected range of machining variables, single-objective optimization results showed that maximum MRR of 0.3228 g/min, minimum SR of 5.94 µm, and minimum RLT of 20.59 µm was obtained. It is evident from the results that with an increase in T_on_, MRR increases, but at the same time SR and RLT also reach a maximum value, which is not desirable. Similarly, minimum SR and RLT were obtained with minimum T_on_ time, but by doing so, MRR is compromised as it gives the least MRR of 0.1988 g/min. Hence, it can be concluded that the response parameters are conflicting and results of the single-objective optimization allow a user to select the machining variables in such a way that either of the response parameters can be maximized or minimized. It is important to solve the trade-off between these variables so that the optimum combination of parametric settings for the WEDM process can be adapted. Such complexity can be resolved by solving the problem using a multi-objective optimization approach. Equal weights of 0.33 have been assigned to the output responses to perform the simultaneous optimization by using the MOTLBO algorithm. Equation (11) shows the objective function for simultaneous optimization, as follows:

(11) $$\mathrm{Obj}\;=w_{1}\cdot \left(\mathrm{MRR}\right)+w_{2}\cdot \left(\mathrm{SR}\right)+w_{3}\cdot \left(\mathrm{RLT}\right)$$

The simultaneous optimization result has yielded response values for MRR, SR, and RLT of 0.2539 g/min, 3.65 µm, and 12.28, respectively, at T_on_ of 31 µs, T_off_ of 12 µs, and Al_2_O_3_ conc. of 2 g/L.

In the basic TLBO algorithm, learners learn from a single teacher and through interaction among themselves, and subsequently, there are two possibilities that either the learner learns completely or nothing at all. The modified algorithm addresses the problems by dividing the learners into small groups and each group of learners is assigned a teacher, thus, having more than one teacher in a class. Now, the teacher has to improve the skills of the respective assigned group of learners and once the desired results are attained, they are allotted to the next best teacher. Another modification in terms of the adaptive teaching factor is incorporated in the basic algorithm. The learners may learn in any proportion from the teacher and not just two or one as in basic algorithm. Such modifications help in a faster convergence rate and speed up exploration and exploitation in the search space.

**Figure 6:**
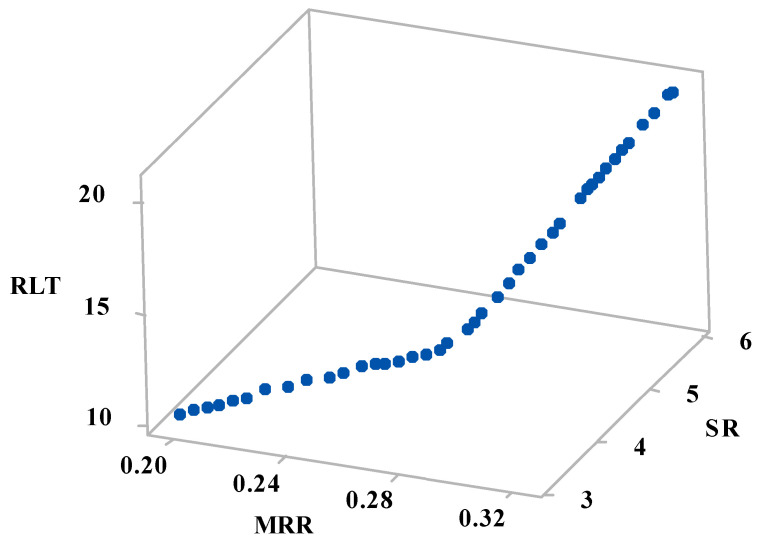
3D Pareto graph of MRR vs. SR vs. Kerf taper angle.

Table 7 shows the 48 Pareto points of multi-objective optimization, where each point represents a unique optimal combination of machining variables between the bounds. The optimal Pareto curve for the three response parameters was plotted in 3D with X, Y, and Z-axis representing MRR, RLT, and SR, respectively, as shown in Figure 6. Each combination of the machining variables selected from the Pareto front will yield optimum machining results, with the intended objective of maximizing MRR and minimizing RLT & SR. The input parameters T_on_, T_off_, and Al_2_O_3_ nanopowder influence the response parameters concerning the magnitude of variables and the selection of the optimum combination of machining variables is left to the user for attaining the desired outcome.

To validate the results of the optimization algorithm, five optimal points are selected from the Pareto front, and machining is carried out to measure the response parameters. Table 8 shows the comparison of predicted values of MRR, SR, and RLT obtained from the MOTLBO algorithm and experimentally measured response values. It was observed that the experimental results are in good agreement with the optimization results and the measured responses are well within 5% variation, which is regarded as a negligible variance.

**Figure 7:**
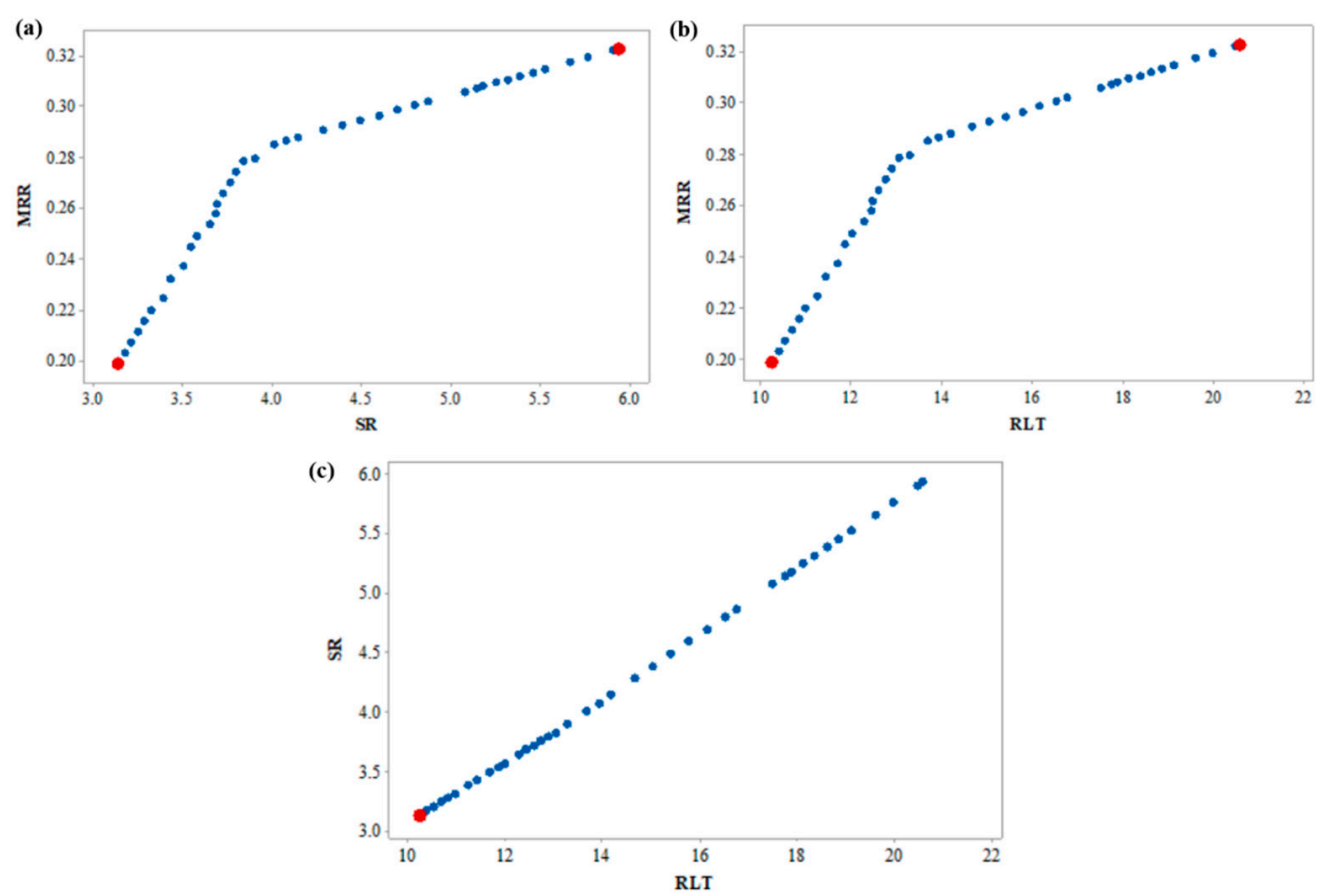
2D Pareto graph (**a**) MRR vs. SR, (**b**) MRR vs. RLT, and (**c**) SR vs. RLT.

To understand the effect of the variables on the response parameters, simplified 2D graphs were plotted considering the effect of the third variable. Figure 7a shows the effect of the machining variables on MRR and SR. It was observed that with an increase in machining MRR, SR increases, resulting in poor machining surface. A maximum MRR (marked with red dot) of 0.322 g/min was obtained with a maximum SR of 5.94 µm, whereas a minimum SR of 3.13 µm was obtained when the MRR was 0.198 g/min. Users can work with the conflicting nature of the parameters and select the combination with the intended objective. Similarly, Figure 7b,c represents the effect of the variables on MRR & RLT and SR & RLT, respectively. Higher MRR results in higher RLT, which is again not a desirable outcome, however, for a user, it is easy to proceed with the combination that can be aimed for either maximum MRR or minimum RLT. However, a linear relationship between SR and RLT is observed indicating that with an increase in SR, RLT increases linearly, and hence, it is always desired to have their minimum. From Figure 7c, it is obvious that minimum SR and RLT (marked with red dot) were obtained as 3.13 µm and 10.24 µm, respectively.

### 4.7. Surface Morphological Study

**Figure 8:**
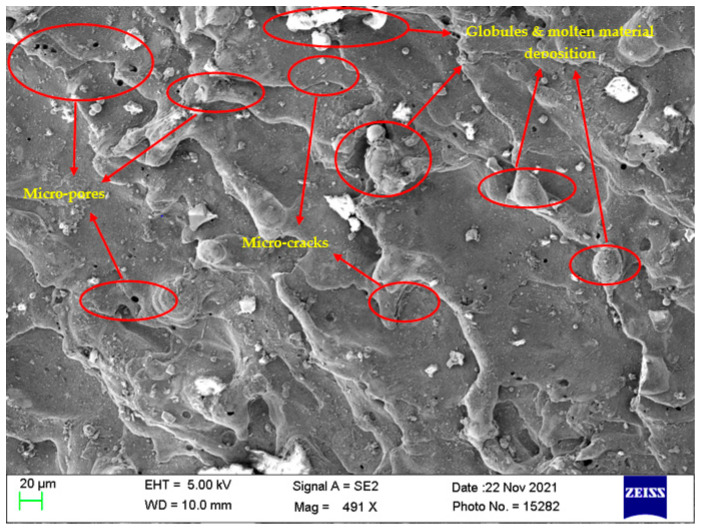
Surface morphology at T_on_ = 31 µs, T_off_ = 12 µs, and Al_2_O_3_ amount = 0 g/L.

**Figure 9:**
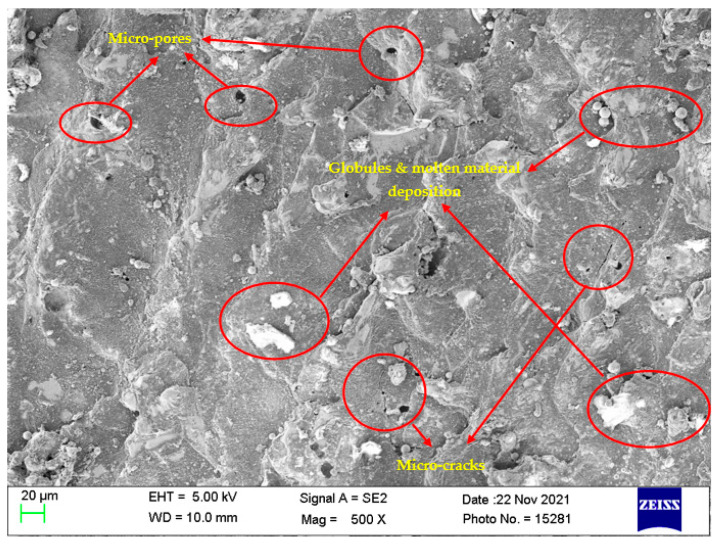
Surface morphology at T_on_ = 31 µs, T_off_ = 12 µs, and Al_2_O_3_ amount = 1 g/L.

**Figure 10:**
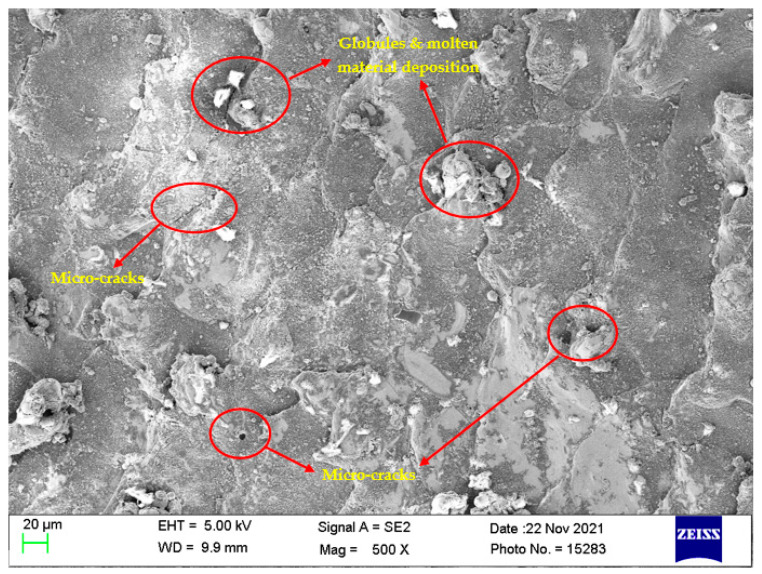
Surface morphology at T_on_ = 31 µs, T_off_ = 12 µs, and Al_2_O_3_ amount = 2 g/L.

Investigation of the machined surfaces was carried out using FE-SEM. Specimens were explored for a surface morphological study through the SEM technique at 500× magnification. The optimal parameter settings obtained through simultaneous optimization of responses (Equation (5)) were used to study the surface morphology of the obtained machined surfaces at different nanopowder concentrations. The simultaneous optimal parameter settings have a T_on_ of 31 µs, T_off_ of 12 µs, and Al_2_O_3_ conc. of 2 g/L. Surface morphology analysis was carried out to find the effect of Al_2_O_3_ conc. According to this, Al_2_O_3_ conc. was varied at 0 g/L, 1 g/L, and 2 g/L, while keeping T_on_ and T_off_ at their optimal values of 31 µs and 12 µs, respectively. Figure 8, Figure 9 and Figure 10 illustrate the SEM micrographs at Al_2_O_3_ conc. Of 0 g/L, 1 g/L, and 2 g/L, respectively. A large number of surface defects can be observed in Figure 8. A large amount of melted material deposition, micro-cracks, globules of debris, and micro-pores was found on the machined surface. Figure 9 (Al_2_O_3_ amount of 1 g/L) shows improved surface in terms of reduction in surface defects in comparison with Figure 8 (Al_2_O_3_ amount of 0 g/L). A substantial improvement can be observed in the surface morphology of Figure 10 (Al_2_O_3_ amount of 2 g/L), which clearly shows a major reduction in surface defects. A large reduction in melted material deposition, micro-cracks, globules of debris, and micro-pores can be seen in Figure 10. This clearly shows that the addition of Al_2_O_3_ amount has reduced the surface defects to a larger extent. The addition of nanopowder creates uniform sparking among the work–tool interface [40,66]. This uniform sparking between work and tool has reduced the micro-crack substantially [67]. The addition of nanopowder concentration expands the interelectrode gap and also increases the heat dissipation in the dielectric fluid by forming small craters and reducing the plasma heat flux [27,31]. In turn, this reduces the surface defects of the melted material deposition, formation of globules, and micro-pores. The addition of Al_2_O_3_ nanopowder enhances the flushing of debris from the machining zone [31]. The improved flushing of debris forms small ridges resulting in improved surface quality.

## 5. Conclusions

In the current study, pulse-on time (T_on_), pulse-off time (T_off_), and Al_2_O_3_ nanopowder concentration were used for achieving higher MRR and lower SR and RLT for the WEDM machining of Nitinol. TLBO algorithm was used to achieve the desired responses simultaneously. The below mentioned useful remarks can be made based on the present study:

- Statistical analysis from ANOVA showed that all WEDM variables (T_on_, T_off_, and Al_2_O_3_ nanopowder) were observed to have a significant effect on all the response variables (MRR, SR, and RLT).
- For MRR, Al_2_O_3_ nanopowder has the highest contributing effect of 76.13%, followed by T_off_ (17.47%) and T_on_ (7.14%). For SR, T_on_ was found to be the highest contributing factor (91.88%), trailed by T_off_ (6.36%) and Al_2_O_3_ nanopowder (1.17%). For RLT, T_on_ was found to be the highest contributing factor (88.3%), trailed by T_off_ (7.85%) and Al_2_O_3_ nanopowder (3.38%). Negligible error contribution was observed for all responses.
- The proposed model can be treated as adequate and the best fit as close relation between R-sq. and Adj. R-sq. was obtained and their values were near to 1. This also signifies that the model is appropriate for predicting the future outcomes of MRR.
- The main effect plots for MRR, SR, and RLT illustrated that the addition of Al_2_O_3_ nanopowder improved the performance of all response variables.
- With the addition of Al_2_O_3_ nanopowder, the rate of erosion of the work material increased and gave an increased MRR. With the addition of Al_2_O_3_ nanopowder, the formation of small craters and uniform flushing of debris resulted in lower SR. With the addition of Al_2_O_3_ nanopowder, redeposition of debris was removed from the machined zone resulting in thin RLT.
- The single-objective optimization result from TLBO yielded maximum MRR of 0.3228 g/min (at T_on_ of 90 µs, T_off_ of 5 µs, and amount of Al_2_O_3_ of 2 g/L), minimum SR of 3.13 µm, and minimum RLT of 10.24 µm (both responses at T_on_ of 30 µs, T_off_ of 25 µs, and amount of Al_2_O_3_ of 2 g/L).
- The simultaneous optimization result yielded response values of MRR, SR, and RLT of 0.2539 g/min, 3.65 µm, and 12.28, respectively, at T_on_ of 31 µs, Toff of 12 µs, and Al_2_O_3_ conc. of 2 g/L.
- A multi-objective TLBO algorithm was used to generate Pareto optimal points highlighting the non-dominant feasible solutions.
- It was observed that the experimental results were in good agreement with the optimization results and the measured responses were well within 5% variation. The least error between predicted and actual values suggests the effectiveness of both the regression model and TLBO algorithm.
- A considerable reduction in surface defects (melted material deposition, micro-cracks, globules of debris, and micro-pores) owing to the addition of Al_2_O_3_ nanopowder (from 0 g/L to 2 g/L) was observed in the surface morphology analysis.

## Figures and Tables

**Table 1:** Experimental conditions of PMWEDM operation.

Working Condition	Description
Pulse-on time (µs)	30, 60, 90
Pulse-off time (µs)	5, 10, 15
Al_2_O_3_ concentration (g/L)	0, 1, 2
Discharge current (A)	3
Graphene nanopowder-size (nm)	100
Powder	Al_2_O_3_
Wire	Molybdenum

**Table 2:** Taguchi’s L_9_ OA and the measured values of MRR, SR, and RLT.

Run	T_on_ (µs)	T_off_ (µs)	Al_2_O_3_ Conc. (g/L)	MRR (g/min)	SR (µm)	RLT (µm)
1	30	5	0	0.084073	4.056	14.160
2	30	15	1	0.114645	3.744	13.120
3	30	25	2	0.127266	3.464	11.728
4	60	5	1	0.155865	4.608	16.240
5	60	15	2	0.171259	4.336	14.576
6	60	25	0	0.070416	4.296	15.000
7	90	5	2	0.201043	5.224	18.328
8	90	15	0	0.087797	5.136	17.904
9	90	25	1	0.117559	5.040	17.584

**Table 3:** ANOVA for MRR.

Source	DF	SS	MS	F	*p*	% Contr.
**Regression**	3	0.014745	0.004915	73.71	0.000	
**T_on_**	1	0.001078	0.001078	16.16	0.010	7.14
**T_off_**	1	0.002635	0.002635	39.52	0.001	17.47
**Al_2_O_3_ Conc.**	1	0.011032	0.011032	165.44	0.000	73.16
**Error**	5	0.000333	0.000067			2.23
**Total**	8	0.015079				

R-Sq. = 97.79%, R-Sq. (Adj.) = 96.82%, R-Sq. (pred.) = 91.78%.

**Table 4:** ANOVA for SR.

Source	DF	SS	MS	F	*p*	% Contr.
**Regression**	3	3.08426	1.02809	275.01	0.000	
**T_on_**	1	2.85108	2.85108	762.67	0.000	91.88
**T_off_**	1	0.19729	0.19729	52.78	0.001	6.36
**Al_2_O_3_ Conc.**	1	0.03588	0.03588	9.60	0.027	1.17
**Error**	5	0.01869	0.00374			0.58
**Total**	8	3.10295				

R-Sq. = 99.40%, R-Sq. (Adj.) = 99.04%, R-Sq. (pred.) = 97.71%.

**Table 5:** ANOVA for RLT.

Source	DF	SS	MS	F	*p*	% Contr.
**Regression**	3	40.7821	13.590	114.25	0.000	
**T_on_**	1	36.5461	36.5461	307.14	0.000	88.3
**T_off_**	1	3.2502	3.2502	27.31	0.003	7.85
**Al_2_O_3_ Conc.**	1	0.9858	0.9858	8.28	0.035	3.38
**Error**	5	0.5949	0.1190			0.47
**Total**	8	41.3770				

R-Sq. = 98.56%, R-Sq. (Adj.) = 97.70%, R-Sq. (pred.) = 95.83%.

**Table 6:** Single-objective optimization results.

Objective Function	T_on_ (µs)	T_off_ (µs)	Powder Conc. (g/L)	MRR (g/min)	SR (µm)	RLT (µm)
Maximum MRR	90	5	2	0.3228	5.94	20.59
Minimum SR	30	25	2	0.1988	3.13	10.24
Minimum RLT	30	25	2	0.1988	3.13	10.24

**Table 7:** Pareto optimal points obtained from HTS algorithm.

Sr. No.	Pulse on Time (µs)	Pulse off Time (µs)	Powder Conc. (g/L)	MRR (g/min)	SR (µm)	RLT (µm)
1	90	5	2	0.3228	5.94	20.59
2	30	25	2	0.1988	3.13	10.24
3	82	5	2	0.3175	5.66	19.60
4	85	5	2	0.3195	5.76	19.97
5	32	6	2	0.2798	3.90	13.29
6	34	5	2	0.2853	4.01	13.68
7	78	5	2	0.3148	5.52	19.11
8	89	5	2	0.3221	5.90	20.47
9	45	5	2	0.2927	4.39	15.04
10	30	17	2	0.2323	3.43	11.42
11	30	23	2	0.2072	3.21	10.54
12	30	24	2	0.2030	3.17	10.39
13	30	13	2	0.2491	3.57	12.01
14	30	8	2	0.2700	3.76	12.75
15	31	19	2	0.2246	3.39	11.25
16	30	7	2	0.2742	3.79	12.89
17	30	6	2	0.2784	3.83	13.04
18	72	5	2	0.3108	5.32	18.37
19	65	5	2	0.3061	5.07	17.51
20	70	5	2	0.3094	5.25	18.12
21	51	5	2	0.2967	4.59	15.78
22	76	5	2	0.3134	5.45	18.86
23	74	5	2	0.3121	5.38	18.62
24	48	5	2	0.2947	4.49	15.41
25	36	5	2	0.2866	4.07	13.93
26	42	5	2	0.2907	4.28	14.67
27	38	5	2	0.2880	4.14	14.17
28	42	5	2	0.2907	4.28	14.67
29	30	10	2	0.2617	3.68	12.45
30	54	5	2	0.2987	4.70	16.15
31	57	5	2	0.3007	4.80	16.52
32	67	5	2	0.3074	5.14	17.75
33	68	5	2	0.3081	5.18	17.88
34	30	20	2	0.2198	3.32	10.98
35	38	5	2	0.2880	4.14	14.17
36	30	20	2	0.2198	3.32	10.98
37	31	12	2	0.2539	3.65	12.28
38	30	21	2	0.2156	3.28	10.83
39	30	9	2	0.2659	3.72	12.60
40	30	14	2	0.2449	3.54	11.86
41	31	11	2	0.2581	3.68	12.43
42	30	21	2	0.2156	3.28	10.83
43	30	22	2	0.2114	3.24	10.68
44	30	22	2	0.2114	3.24	10.68
45	30	14	2	0.2449	3.54	11.86
46	30	9	2	0.2659	3.72	12.60
47	31	16	2	0.2372	3.50	11.69
48	59	5	2	0.3020	4.87	16.77

**Table 8:** Confirmatory trials.

Sr. No.	T_on_ (µs)	T_off_ (µs)	Powder (g/L)	Prediction from TLBO			Actual Experimental Values			% Error		
				MRR	SR	RLT	MRR	SR	RLT	MRR	SR	RLT
1	90	5	2	0.3228	5.94	20.59	0.3381	5.73	20.88	4.52	3.66	1.38
2	30	25	2	0.1988	3.13	10.24	0.2073	3.11	10.11	4.10	0.64	1.28
30	54	5	2	0.2987	4.70	16.15	0.2892	4.92	16.67	3.28	4.47	3.11
37	31	12	2	0.2539	3.65	12.28	0.2499	3.78	11.98	1.60	3.43	2.5
47	31	16	2	0.2372	3.50	11.69	0.2432	3.41	12.01	2.46	2.63	2.66

## Data Availability

Data presented in this study are available in this article.
